# Broadband metasurface superstrate for polarization-independent wave focusing and gain enhancement at Ka-band

**DOI:** 10.1038/s41598-022-16037-1

**Published:** 2022-07-14

**Authors:** Kanghyeok Lee, Ha Young Hong, Wonwoo Lee, Semin Jo, Hong Soo Park, Junhyuk Yang, Changkun Park, Hojin Lee, Sun K. Hong

**Affiliations:** 1 Department of Information Communication Convergence Technology, Soongsil University, Seoul, 0678 Korea; 2 School of Electronic Engineering, Soongsil University, Seoul, 0678 Korea

**Keywords:** Metamaterials, Electrical and electronic engineering

## Abstract

A broadband metasurface flat lens is proposed as a polarization-independent wideband superstrate for wave focusing and gain enhancement at Ka-band. The proposed metasurface structure consists of four metal layers and is designed with diagonally symmetric unit cells to accommodate both the vertical and horizontal polarizations. The focusing ability of the proposed metasurface flat lens is validated via simulation and measurement, where normally incident plane waves are shown to be enhanced by up to 11 dB as a result of wave focusing. Also, the radiation gain enhancement due to the proposed metasurface flat lens is demonstrated via simulation and measurement, where a gain enhancement of up to 10.5 dB is achieved. The results show that the proposed structure maintains the wave focusing and gain enhancement characteristics over a bandwidth of 28–32 GHz. Furthermore, to demonstrate the utility of the proposed metasurface for circular polarization (CP), the gain enhancement of a CP patch antenna as a result of implementing the proposed metasurface as a superstrate is demonstrated via simulation and measurement. It is shown that the proposed metasurface superstrate provides a CP gain enhancement of nearly 10 dB.

## Introduction

Research on wireless power transfer (WPT) using millimeter waves (mm-waves) has gained considerable attention in recent years^1–5^. Due to their shorter wavelengths, mm-waves offer superior directionality than microwaves and allow for physically compact systems. It means that Ka-band WPT systems can be more readily accommodated in compact electronics such as mobile, portable devices, and small drones, thereby overcoming spatial constraints. However, mm-waves suffer from higher path loss, atmospheric and dielectric attenuation compared to microwaves in performing wireless power transfer. To overcome such drawbacks, the overall WPT efficiency from transmitter to receiver must be improved. On the transmitting end, beamforming^6–9^ is generally used to direct a narrow beam, thereby concentrating electromagnetic power at a device of interest and improving efficiency. On the receiving end, the efficiency can be improved by increasing the aperture efficiency and gain of the receive antenna and/or increasing the rectification efficiency of the RF-to-DC rectifier.

Here, our focus is on the design of a receiving structure that offers high aperture efficiency. Hence, metasurface lens^10^, a thin two-dimensional form of metamaterials in which subwavelength unit cells are periodically arranged, is proposed as a method to produce the enhanced gain. Metasurface lens controls the amplitude and phase of the incident plane wave in each unit cell. By employing it as a superstrate for a receive antenna, it offers higher aperture efficiency with lightweight and compact size than conventional array antennas^11–26^. For an effective WPT system, phase gradient metasurfaces in a flat lens type are applied to the receiving end to be utilized as a superstrate of an antenna. A flat metasurface lens with low loss can be designed to reduce volume while ensuring wide phase-shift coverage and wave focusing to enhance the radiation performance of the antenna^27–41^. In particular, wideband and polarization independence are important features to facilitate mm-wave WPT for various incident waves.

In this paper, we propose the design of a wideband, polarization-independent metasurface flat lens that can be used as a superstrate of the receive antenna in a wireless power transfer system at Ka-band. In particular, polarization independence is an important feature for receiving wireless power, since the polarizations of the transmit and receive antennas are prone to be misaligned in practice. Due to its wide operating frequency bandwidth ranging from 28 to 32 GHz, the proposed structure can potentially be applied to systems operating at various Ka-band frequencies. The focusing ability and gain enhancement of the proposed lens are demonstrated via simulation and measurement. The polarization independence is also verified using the proposed metasurface as a superstrate of a circularly polarized patch antenna.

## Metasurface flat lens design

**Figure 1:**
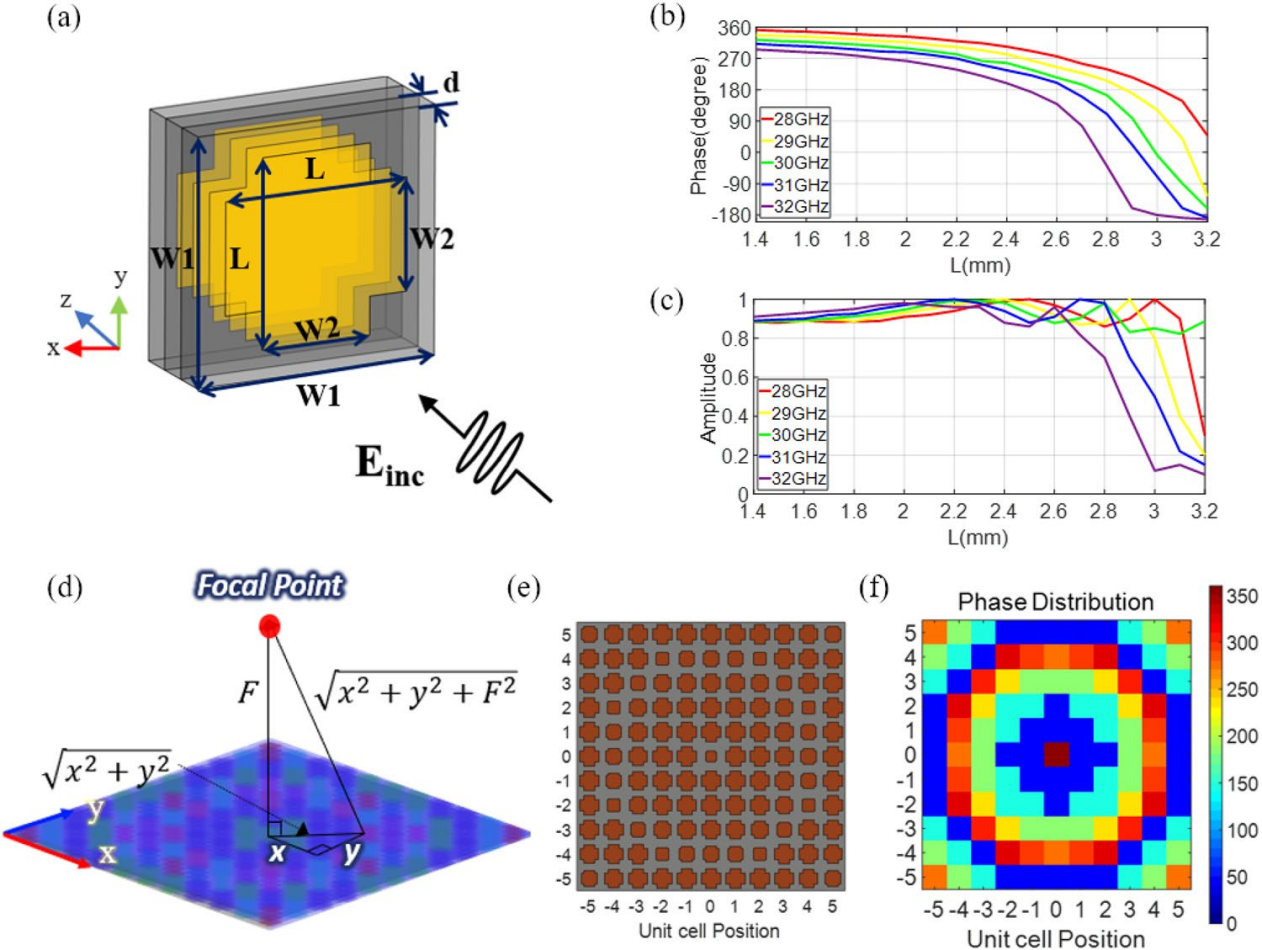
(**a**) Unit cell design of the proposed metasurface. *W1* = 4 mm, *W2* = 1.8 mm, *d* = 0.5 mm. The simulated transmission coefficient of the metasurface for various values of *L* at different frequencies. (**b**) Phase and (**c**) amplitude. (**d**) Illustration of the distance from the center coordinate and arbitrary coordinate of the lens to the focal point. (**e**) The unit cell configuration of the metasurface lens. (**f**) Calculated phase distribution at each unit cell.

A unit cell of the proposed metasurface structure, as shown in Fig. 1a, comprises four identically shaped metal layers separated by three dielectric substrates. The number of layers is chosen to ensure a wide operating bandwidth. A Rogers RT/Duroid 5870 (relative permittivity = 2.33) laminate is used as the substrate with a thickness of 0.5 mm. When a normal incident plane wave passes through the unit cell, the intensity and phase of the incident wave change depending on the size of the metal patch. The metal part is designed in the shape of a diagonally symmetric thick cross to obtain polarization-independent characteristics. A thick rectangular shape with a length *L* and width *W2* is used to cover a single polarization. Then, the rectangular shape is rotated 90 degrees and overlapped to form a thick cross shape for the orthogonal polarization. The values of *L* and *W2* are important in determining the phase and amplitude changes of incident plane waves. To configure the unit cells in a planar array formation, each unit cell in the array should accommodate the phase change necessary to focus waves while maintaining high transmissivity. It means that the values of *L* and *W2* in each unit cell must be carefully optimized to cover the phase in [0°, 360°] range.

A full-wave simulation of the unit cells having various values of *L* and *W2* is conducted using SEMACAD X^42^. As shown in Fig. 1a, the phase and amplitude of a plane wave normally incident on the unit cell are probed after passing through the unit cell. Figure 1b,c show the phase and amplitude of the transmission coefficient according to various values of *L* at different frequencies. To cover a full 360° phase change in the desired frequency band, the value of *L* that determines the phase change needs to be tuned between 1.4 mm and 3.2 mm. It is demonstrated that the values of *L* in the chosen range can produce phase changes over [0°, 360°] at the frequencies of interest including the center frequency of 30 GHz. The minimum allowable transmission amplitude is set to 0.8, and it is confirmed that when the value of *W2* is 1.8 mm, it would generally produce the transmission amplitude of at least 0.8 over the frequency range of interest. In Fig. 1c, it is shown that the transmission amplitude maintains 0.8 or higher at the center frequency for all values of *L*. At other frequencies in the band, the transmission amplitude generally remains above 0.8 except for a few sets of values of *L*. Based on these results, the overall planar size of the unit cell (*W1* × *W1*) is designed to be 4 × 4 mm^2^, which corresponds to 0.4 λ × 0.4 λ at the center frequency, 30 GHz.

The overall metasurface flat lens is designed by an array of the proposed unit cells. Each unit cell manipulates the phase changes by its position in the planar lens. The phase at each unit cell location is determined by different path lengths between the focal length *F* and the distance from the focal point to the unit cell ($\sqrt{{\mathrm{x}}^{2}+{y}^{2}+{F}^{2}}$), as illustrated in Fig. 1d. The phase distribution on the metasurface lens follows Eq. (1) based on Fermat’s principle^43^ to compensate for path differences.

1 $$\varphi \left(m,n\right)={k}_{0} \left(\sqrt{{F}^{2}+{\left(mW1\right)}^{2}+{\left(nW1\right)}^{2}}-F \right),$$

where *m* and *n* respectively refer to the position number of the unit cells in the x- and y-axis, and *k*_o_ is the propagation constant at the center frequency of 30 GHz. Here, the focal length *F* is set to 16 mm, which corresponds to nearly one-half of a wavelength at the center frequency. Hence, the value *L* in each unit cell can be configured using Fig. 1b according to the obtained phase *φ* of each unit cell by Eq. (1). Based on the optimized parameters, the metasurface flat lens is designed by arranging the unit cells into an 11 × 11 array, overall planar dimensions of the metasurface 44 × 44 mm^2^ (4.4 λ × 4.4 λ at 30 GHz) as shown in Fig. 1e. The resulting phase distribution in each of the 121 unit cells of the metasurface structure is shown in Fig. 1f.

## Results

The performance of the proposed metasurface flat lens is tested via simulation and measurement. As mentioned in the introduction, the purpose of this metasurface flat lens is to be used as a superstrate of a receive antenna in wireless power reception. As such, the test is done in two different experiments. The first experiment is to verify the focusing ability and focal point where the electric field enhancement is greatest. In this case, the field intensity behind the lens (the region after the incident waves have passed through the metasurface) is measured through a planar near-field scan. The second experiment is for the gain enhancement validation, performed by measuring the radiation pattern of a single antenna element with and without the metasurface lens to determine the gain enhancement resulting from wave focusing.

### Focusing results

**Figure 2:**
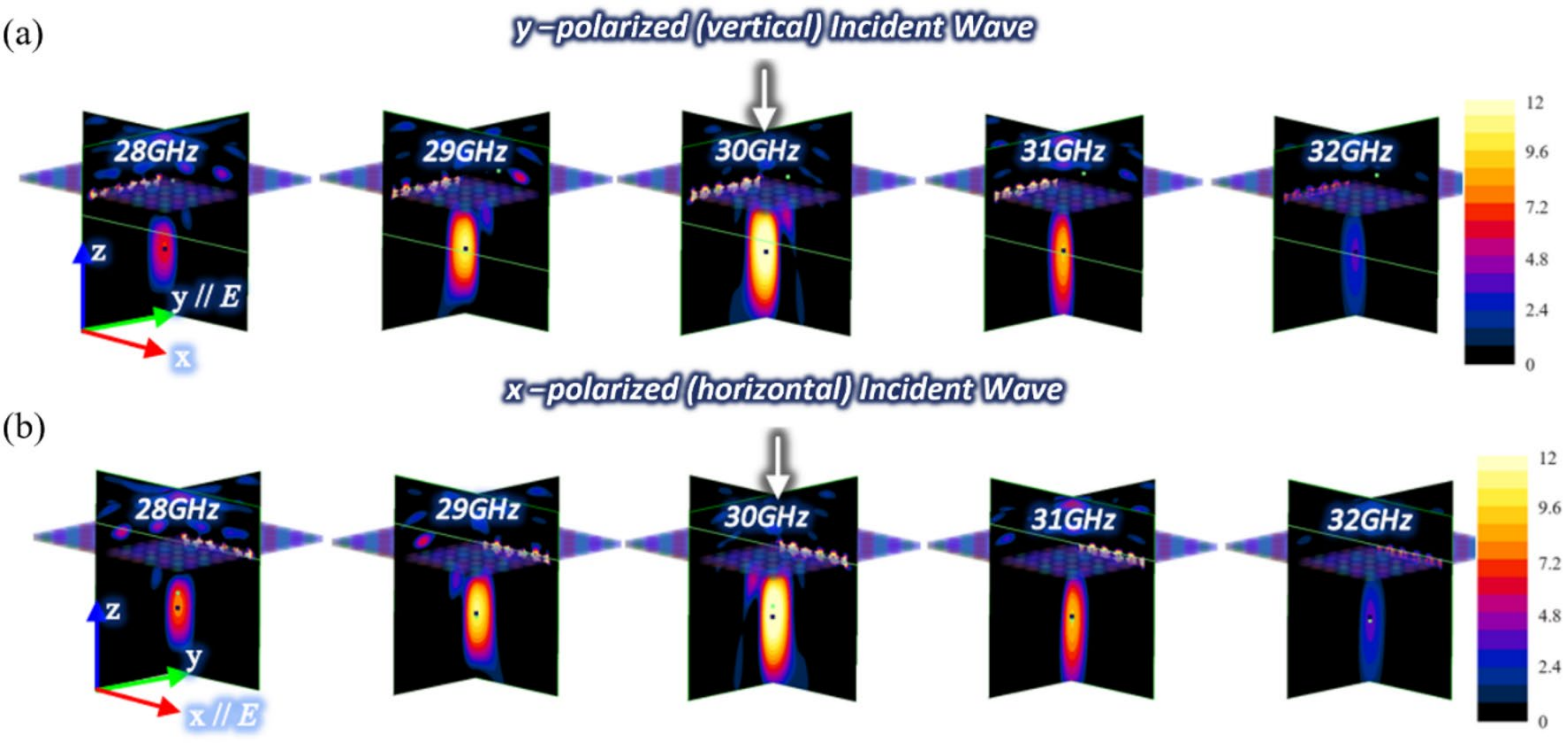
Simulated field intensity distribution for (**a**) y-polarized (vertical) and (**b**) x-polarized (horizontal) incident plane waves at various frequencies.

Figure 2a,b show the simulated electric field intensity at various frequencies within 28–32 GHz after a normally incident plane wave has passed through the proposed metasurface flat lens. In the figure, the electric field intensity in the x–z and y–z planes are shown. In Fig. 2a, the fields resulting from a y-polarized (vertical) incident plane wave are shown, while in Fig. 2b, the fields resulting from an x-polarized (horizontal) incident plane wave are plotted. Note that the field intensity values are plotted in dB with respect to the incident plane wave field value. For both polarizations, wave focusing is observed around the targeted focal point, that is, 16 mm away from the center of the metasurface. The strongest focus occurs at the center frequency with the electric field enhancement of 11 dB.

**Figure 3:**
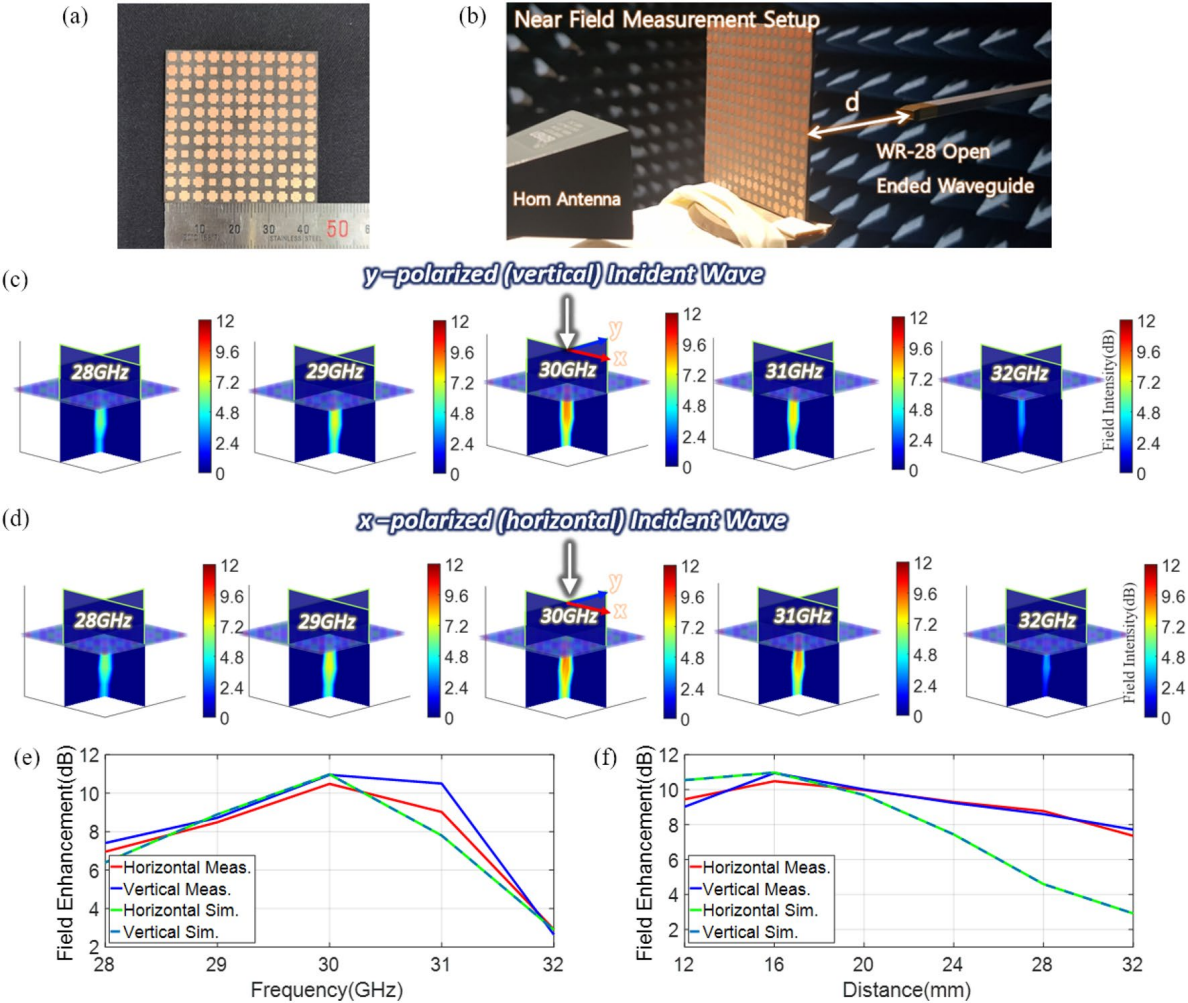
(**a**) Photographs of a fabricated metasurface lens. (**b**) Near-field scan setup for measuring the field intensity passing through the metasurface lens. Measured field distribution for (**c**) y-polarized (vertical) and (**d**) x-polarized (horizontal) incident plane waves at various frequencies. Simulated and measured field enhancement vs. (**e**) frequency when 16 mm apart from the center of the metasurface lens and (**f**) distance from the center of the lens when the frequency is 30 GHz.

To validate the simulated focusing results, a near-field scan measurement is performed in an anechoic chamber. Figure 3a shows the front view of the fabricated metasurface flat lens and Fig. 3b shows the measurement setup. A standard gain horn antenna is used to radiate normally incident plane waves at the metasurface. In the actual measurement, the horn antenna is placed farther (at a far-field distance) than it is shown in the figure. For near-field probing, a WR-28 open-ended waveguide antenna and a planar positioner are used to scan along the x–z and y–z planes. The scan is performed over an area of 50 × 50 mm^2^ in each plane. Both the vertical and horizontal polarizations are measured by rotating the horn antenna and waveguide probe antenna by 90 degrees in each scan. Figure 3c,d show the measured field intensity in both the x–z and y–z planes for vertical and horizontal polarizations, respectively. The field intensity is a relative quantity in dB with respect to the incident plane wave measured without placing the metasurface lens. Similar to the simulated results, the measured values show the field intensity at various frequencies within 28–32 GHz. In the measured results, it is clearly observed that wave focusing occurs around the intended focal point of 16 mm. Furthermore, the strongest focus takes place at the center frequency with the electric field enhancement of 11 dB and 10.5 dB for vertical and horizontal polarizations, respectively. Overall, the measured and simulated results are in good agreement.

Figure 3e,f summarize some key results obtained in Fig. 3c,d. Figure 3e is a plot showing the electric field enhancement as a function of frequency at the focal point. The simulated and measured values show a very similar trend, where the strongest field enhancement occurs at 30 GHz, while a reasonable enhancement is also seen at other frequencies for both vertical and horizontal polarizations. Figure 3f is a graph of the electric field enhancement as a function of the distance (along the z-axis) from the center of the metasurface lens at 30 GHz. The plot shows the simulated and measured values at the various distance at an interval of 4 mm. In both measured and simulated results, a maximum field enhancement occurs at the intended focal point for both polarizations and falls off as the distance increases. The measured values, however, seem to decrease a bit slower than the simulated values, implying that after focusing, the fields diverge slower in the measured case. Nevertheless, a similar general trend is observed for both measured and simulated results, validating the focusing performance of the proposed metasurface lens.

### Gain enhancement results

**Figure 4:**
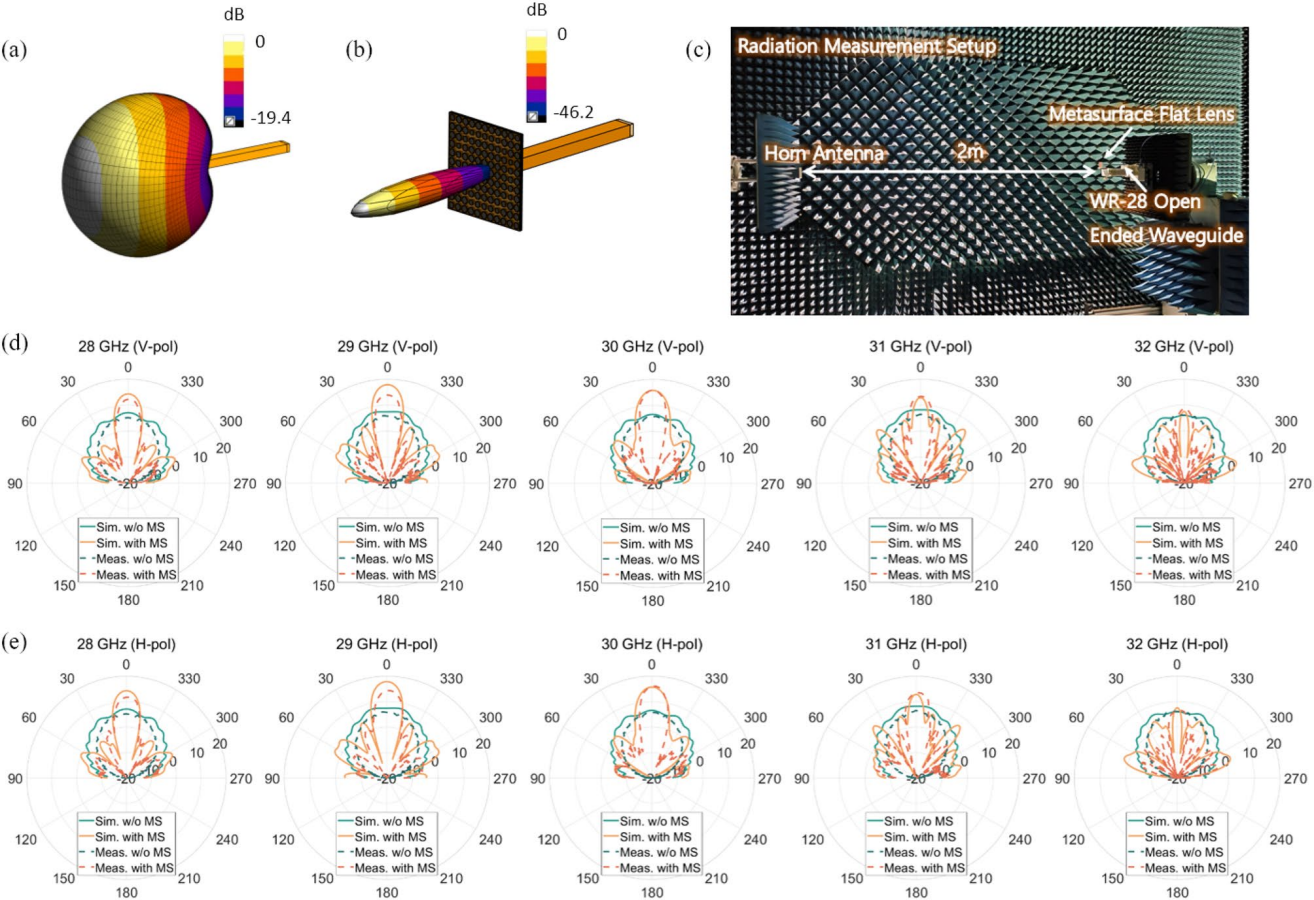
The simulated 3-D radiation pattern of (**a**) WR-28 waveguide probe antenna and (**b**) waveguide probe antenna with the proposed metasurface lens. (**c**) Photographs of measurement setup of gain enhancement of the metasurface lens. Comparison of simulated and measured antenna gain according to the presence or absence of the metasurface lens for (**d**) y-polarized (vertical) radiation and (**e**) x-polarized (horizontal) radiation.

In the radiation experiment, the proposed metasurface is placed in front of a single element antenna to test the radiation gain enhancement. The antenna used in this experiment is the same WR-28 open-ended waveguide antenna used for the nearfield scan measurement. In this case, the metasurface lens is placed at a fixed distance of 16 mm from the antenna, which corresponds to the focal length of the lens, representing a case where the metasurface lens is used as a superstrate of a single element antenna. In practice, the choice of a single element antenna is not limited to an open-ended waveguide. More practically suitable antennas such as the patch or planar antennas may be used. The simulated radiation pattern of the open-ended waveguide probe antenna without the metasurface lens is shown in Fig. 4a. In comparison, the radiation pattern when the metasurface lens is added as a superstrate is plotted in Fig. 4b. It can be seen that the radiation pattern with the metasurface lens is narrower, indicating that the gain is enhanced. To validate the simulated gain enhancement, measurement is performed in an anechoic chamber as shown in Fig. 4c. A standard gain horn antenna is used as the receive antenna at a far-field distance. The radiation pattern in azimuth is measured using a rotating positioner on which the metasurface and antenna are placed. Also, measurements are made for both vertical and horizontal polarization by rotating the open-ended waveguide and the horn antenna by 90 degrees for the respective polarization.

**Figure 5:**
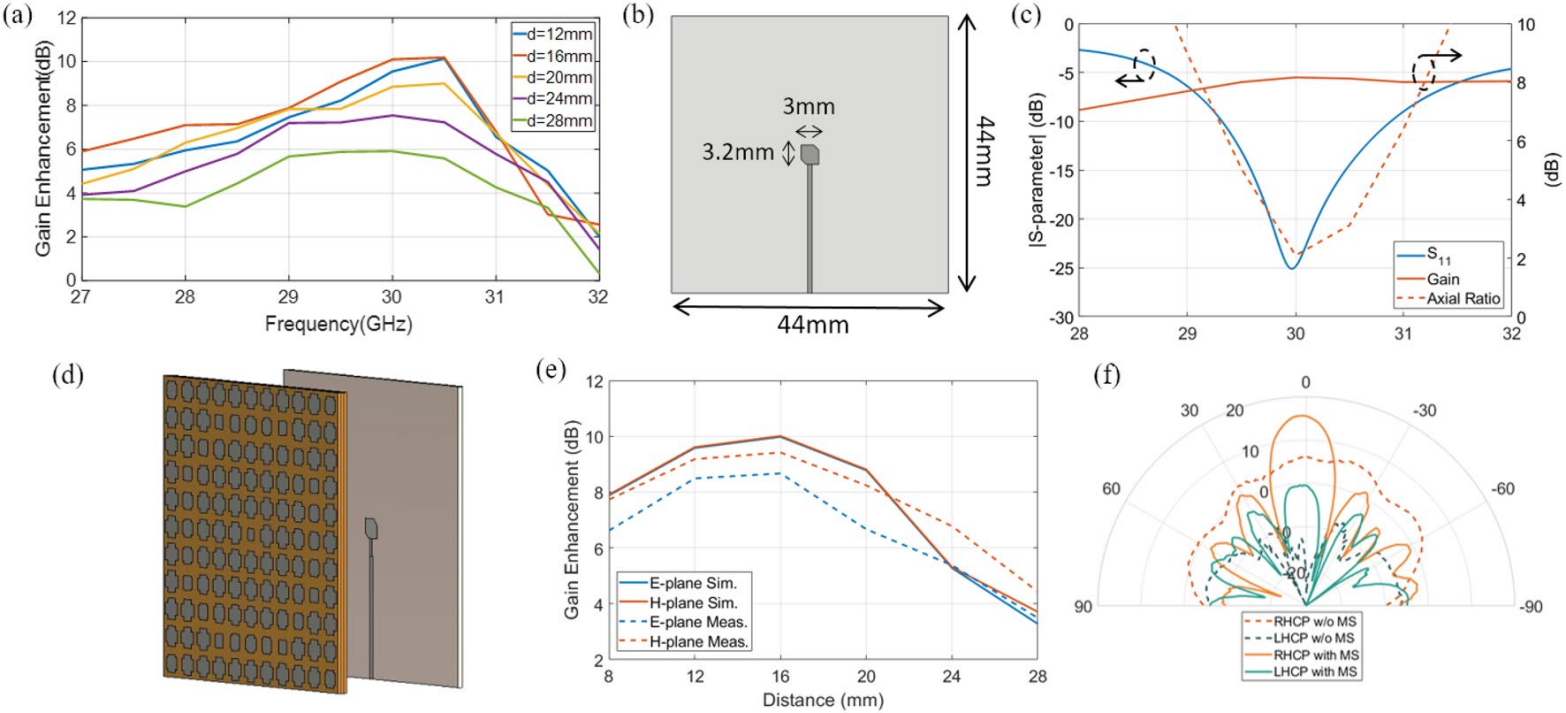
(**a**) Measured antenna gain enhancement of the metasurface superstrate according to the frequency and distance. (**b**) Designed circularly polarized patch antenna with its dimension. (**c**) The characteristics of the designed patch antenna including reflection coefficient, simulated gain and axial ratio. (**d**) Illustration of the metasurface superstrate using the proposed CP patch antenna. (**e**) Simulated and measured results of gain enhancement according to the distance at 30 GHz. (**f**) The measured radiation pattern of the CP patch antenna with and without metasurface lens when 16 mm apart from the lens.

Figure 4d,e show the simulated and measured radiation patterns of the antenna with and without the metasurface lens at various frequencies within 28–32 GHz. In Fig. 4d, the radiation patterns of vertical polarization are shown, while in Fig. 4e, the radiation patterns of the horizontal polarization are plotted. The azimuth scan is performed over a range of *ϕ* = [− 90°, 90°], which is the maximum scan range of the rotating positioner used in the measurement. Simulated and measured results show that the antenna gain is enhanced for both polarizations when the metasurface flat lens is used as a superstrate. The greatest antenna gain enhancement occurs at the center frequency of 30 GHz for both polarizations. For vertical polarization, the simulated and measured gain enhancement at the center frequency is 11.2 dB and 10.3 dB, respectively. For horizontal polarization, the simulated and measured gain enhancement at the same frequency is 11.2 dB and 10.5 dB. The gain improvement results indicate that the maximum field focusing occurs at 30 GHz, as in the case of the field focusing measurement. Furthermore, other frequencies also exhibit gain enhancement values ranging between 3 and 10 dB, which are also in line with the field focusing results. Through further design optimization, the gain enhancement at other frequencies can be improved. The above gain enhancement results are obtained when the distance between the metasurface flat lens and the antenna is 16 mm apart. To verify the maximum focusing distance between the metasurface lens and the antenna related to the gain enhancement, the gain enhancement is measured at various distances (12–28 mm). As shown in Fig. 5a, it is observed that the highest gain enhancement occurs at 16 mm (the focal length of the lens), validating that it is the optimal spacing for the metasurface to be used as a superstrate.

### Polarization independence validation using a CP antenna

Although the aforementioned measurements of vertical and horizontal polarizations demonstrate the polarization independence of the metasurface lens, it would be of interest to verify the practical utility of the proposed metasurface’s polarization independence. To this end, the performance of the proposed metasurface as a superstrate of a circularly polarized (CP) antenna is tested via simulation and measurement. A CP patch antenna is designed as shown in Fig. 5b. The substrate of the CP patch antenna is the same size as the metasurface lens to configure a stable structure with the lens as a superstrate. An RT/Duroid 5880 (relative permittivity = 2.2) laminate is used as the substrate of the CP patch with a thickness of 0.508 mm. The patch has 3.2 mm length and 3 mm width, with a pair of 0.7 mm truncated edges symmetrically to produce right-hand CP radiation. From the simulation, the CP patch antenna shows a 29.3–30.9 GHz bandwidth and 2.03 dB axial ratio at 30 GHz, as shown in Fig. 5c. The gain versus the frequency is also plotted in Fig. 5c. A single CP patch antenna shows a simulated and measured gain of 8.12 and 7.26 dBi at 30 GHz.

The proposed metasurface lens is set to have a distance of 8–28 mm from the designed CP patch antenna to study the performance of gain enhancement, as shown in Fig. 5d. Similar to the results using a waveguide antenna, the simulated and measured results show the maximum enhanced CP antenna gain of 10.01 and 9.42 dB at a 16 mm distance, as shown in Fig. 5e. The enhanced radiation pattern of the measured right-hand CP and left-hand CP at a 16 mm distance are also plotted in Fig. 5f, compared with the pattern of a single patch antenna. It confirms that the metasurface lens can be used independently of polarization, including CP applications. In addition, since the CP patch antenna has a 3 dB beamwidth of 60°, the metasurface flat lens covers not only normal incident waves but also oblique incident waves.

Table 1 shows the performance of the related metasurface lenses reported in the literature in comparison with the proposed metasurface lens. The table compares the frequency band, enhanced gain, unit cell size, overall lens size, focal length, aperture efficiency and characteristics. The aperture efficiency ${\varepsilon }_{ap}$ of the metasurface lens can be calculated as ${\varepsilon }_{ap}=G{\lambda }_{0}^{2}/4\pi {A}_{p}\times 100\%$, where *G* is the maximum gain and *A*_*p*_ is the physical aperture of the metasurface. Compared with the other reported structures in the references, the proposed metasurface flat lens has all of the polarization-independent, wideband, and high gain enhancement characteristics. Therefore, the use of the proposed metasurface flat lens as a superstrate of an antenna can ensure wide phase shift coverage and wave focusing ability, thereby improving antenna gain independently of polarization.

**Table 1:** Comparison of the proposed metasurface lens with referenced designs.

Reference (publication, year)	Frequency (GHz)	Bandwidth (%)	Gain enhancement (dB)	Unit-cell size (λ^2^)	Overall size (λ^2^)	Number of metal layers	Focal length (λ)	Aperture efficiency (%)	Characteristics
TAP, 2015^28^	9.86–10.16	3	11.6	0.33 $\times$ 0.33	4.29 $\times$ 4.29	4	1	30	Polarization-independent
TAP, 2017^29^	9.9–10.2	3	9.2	0.27 $\times$ 0.27	3.46 $\times$ 3.46	2	1	24.6	Polarization-independent
TAP, 2017^30^	6.1–7/9.8–11	13.7/11.5	13.2/13.8	0.24 $\times$ 0.37/0.39 $\times$ 0.6	4.59 $\times$ 4.81/6.63 $\times$ 7.8	4	1.6/2.7	30.3/32.8	Dual-band
ACCESS, 2019^31^	9.4–10.8	13.9	11.3	0.36 $\times$ 0.36	4.68 $\times$ 4.68	4	1.13	35.48	Wideband
TAP, 2020^32^	24–28	15.3	10	0.45 $\times$ 0.45	14.85 $\times$ 14.85	2	15.5	42.25	Wideband
This work	28–32	13.3	10.5	0.4 $\times$ 0.4	4.4 $\times$ 4.4	4	1.6	30	Wideband, polarization independent

## Conclusion

A wideband, polarization-independent Ka-band metasurface flat lens is proposed for use as a superstrate of an antenna to enhance radiation gain as a result of wave focusing. Through simulation and measurement, the performance of the proposed metasurface lens is verified for vertical and horizontal polarization with an operating frequency bandwidth of 28–32 GHz. The field and gain enhancement of up to 11 dB and 10.5 dB, respectively, are obtained. A CP patch antenna in combination with the metasurface superstrate is also demonstrated to validate its practical utility for CP. By implementing the proposed metasurface in the receiving antenna of a Ka-band WPT system, wireless power reception efficiency can be enhanced. Moreover, this device can also be utilized for other Ka-band use such as 5G communications and military applications.

## Data Availability

The datasets used and/or analyzed during the current study available from the corresponding author on reasonable request.
